# A greater number of dissected lymph nodes is associated with more favorable outcomes in bladder cancer treated by radical cystectomy: a meta-analysis

**DOI:** 10.18632/oncotarget.11343

**Published:** 2016-08-17

**Authors:** Fei Li, Xuwei Hong, Lina Hou, Fengsheng Lin, Pengliang Chen, Shiyu Pang, Yuejun Du, He Huang, Wanlong Tan

**Affiliations:** ^1^ Department of Urology, Nanfang Hospital, Southern Medical University, Guangzhou, Guangdong 510515, P. R. China; ^2^ Department of Healthy Management, Nanfang Hospital, Southern Medical University, Guangzhou, Guangdong 510515, P. R. China; ^3^ Department of Urology, The Third People's Hospital of Hubei Province, Wuhan, Hubei 430415, P. R. China

**Keywords:** bladder cancer, lymph node dissection, radical cystectomy, outcome, meta-analysis

## Abstract

The optimal extent of lymph node dissection (LND) is currently not established, and the debate regarding the association between the number of dissected nodes and the outcomes of bladder cancer treated by radical cystectomy (RC) is still ongoing. Therefore, the present meta-analysis was performed to clarify this potential relationship. Eligible studies were retrieved via an electronic search for studies published up to April 2016, and by manual review of the references. A total of 25 cohort studies involving 41,400 bladder cancer patients who underwent RC were included. The summary relative risk estimates (SRRE) based on the highest compared with the lowest categories of LND were estimated by variance-based meta-analysis. Heterogeneity among the study results was explored through stratified analyses. Overall, bladder cancer patients with the highest category of LND had 28%, 34% and 36% reduced risks, corresponding to overall survival (SRRE = 0.72; 95% CI, 0.64–0.80), cancer-specific survival (SRRE = 0.66; 95% CI, 0.54–0.80) and recurrence-free survival (SRRE = 0.64; 95% CI, 0.50–0.82), respectively, compared with patients with the lowest category of LND. In summary, the patients with a greater number of dissected lymph nodes had statistically significant survival advantages in terms of the outcomes of bladder cancer following RC. The number of dissected lymph nodes could be an independent prognostic factor for bladder cancer. These findings need to be validated in prospective and larger epidemiological studies with a longer follow-up period.

## INTRODUCTION

Worldwide, bladder cancer is among the most common malignancies of the genitourinary tract, particularly for men, in whom the incidence of this disease is three- to five-times greater than in women [1]. Approximately 70–80% of new cases are diagnosed as non-muscle-invasive or superficial bladder cancer. Furthermore, > 50% of these cancers will recur, despite treatment by transurethral resection combined with intravesical chemotherapy, and 10–20% of recurrent tumors eventually progress to muscle invasive tumors [2]. At present, radical cystectomy (RC) with pelvic lymph node dissection (LND) is the gold standard for high-risk non-muscle-invasive and muscle-invasive bladder cancer. However, advances in this therapeutic strategy are still associated with unfavorable clinical outcomes and have a limited effect on increasing survival rate [3]. Currently, the stage and grade of tumors are used as the major prognostic factors for these patients, but there is growing interest in identifying additional prognostic indicators to aid medical professionals in improving prognostic evaluations [4].

RC and pelvic LND are important approaches used in the management of muscle-invasive bladder cancer, and LND is considered to be one of the most important steps of the surgery [5]. The number of dissected nodes is an important factor in determining an accurate nodal status; however, there is still an ongoing debate regarding the optimal number of nodes that should be dissected during RC [4]. The potential correlation between the number of dissected nodes and the outcomes of bladder cancer treated with RC has received much attention since 2000 [6–10]; however, a consensus has still not been reached, and a comprehensive assessment concerning the association has not been conducted.

In response, the current study presents the first meta-analysis performed to clarify the potential association between the number of dissected nodes and the outcomes of bladder cancer treated with RC, on the basis of findings from all published epidemiological studies.

## RESULTS

### Literature search results

**Figure 1:**
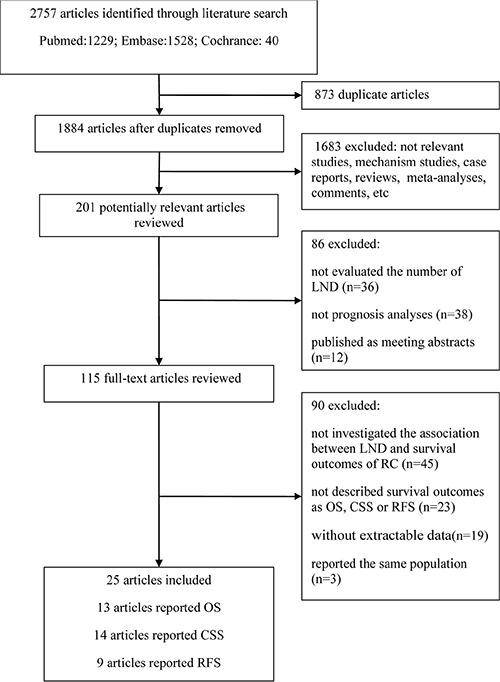
Flow chart for the selection of studies

Figure 1 shows a flow chart of the selection process used in the present study. A total of 2,757 articles were identified via our search strategy. Of the identified articles, 201 were selected for further review following exclusion of duplicate articles and an initial screening of the titles and abstracts. Furthermore, 86 articles were excluded because they did not evaluate the number of dissected lymph nodes, were not prognostic analyses, or were only published as meeting abstracts. After assessing the remaining 115 articles by full-text review, 90 articles were excluded: 45 articles were excluded as they did not investigate the association between the number of dissected nodes and the survival outcomes of bladder cancer after RC; 23 articles were excluded because they did not describe survival outcomes as overall survival (OS), cancer-specific survival (CSS), or recurrence-free survival (RFS); 19 articles were excluded as the number of nodes excised at LND was expressed as a continuous variable; and the other 3 articles were excluded as its participants overlapped with another study [11–13]. Finally, a total of 25 articles were included in the present meta-analysis (Figure 1).

### Characteristics of the studies

The characteristics of the included studies are presented in Table 1. All the included studies were published between 2003 and 2015. The mean period of follow-up ranged from 6–120 months. Of these 25 studies, 10 were conduced in the United States of America [8, 14–22], 4 in Japan [10, 23–25], 2 in Canada [6, 26], 2 in German y [7, 27], 2 in Turkey [9, 28], 2 in international centers [29, 30], and one each in Italy [31], Denmark [32] and Korea [33]. The majority of the included studies used a retrospective design. The meta-analysis comprised a total sample size of 41,400 bladder cancer patients who underwent RC, of which 6,044 patients were reported to have lymph node metastasis.

**Table 1:** Characteristics of studies included in the meta-analysis of the number of dissected lymph nodes and outcomes of bladder cancer following radical cystectomy

Author	Country	Period	Follow-up (months)	Sample size	Mean age (years)	T stage (number of cases)	Extent of LND (high vs. low)	Outcomes of bladder cancer		
								OS	CSS	RFS
Ugurlu 2015 ^[31]^	Turkey	2005~2009	44.9	85	61.0	≤ T2: 42 ≥ T3: 43	≥ 20 vs. < 20	1.17 (0.76–1.81)		1.38 (0.91–2.10)
Siemens 2015 ^[6]^	Canada	1994~2008	NR	1443	69.1	≤ T2: 382 ≥ T3: 1061	> 13 vs. < 5	0.75 (0.64–0.89)	0.74 (0.61–0.89)	
Kang 2015 ^[36]^	Korea	1999~2012	38.0	385	66.0	≤ T2: 246 ≥ T3: 139	≥ 20 vs. < 20	0.41 (0.23–0.71)	0.47 (0.25–0.85)	
Zehnder 2014 ^[32]^	International centers	NR	110.4	521	66.9	≤ T2: 134 ≥ T3: 387	≥ 20 vs. < 20	0.66 (0.52–0.84)		0.71 (0.59–0.92)
Ploussard 2014 ^[33]^	International centers	1979~2012	32.0	8141	68.0	≤ T2: 4624 ≥ T3: 3517	≥ 20 vs. < 20	0.90 (0.84–0.96)		
Lin 2014 ^[17]^	USA	1990~2010	65.9	196	68.0	NR	≥ 20 vs. < 20			0.37 (0.14–1.02)
Gray 2014 ^[18]^	USA	1998~2009	43.0	16,953	67.0	≤ T2: 1525 ≥ T3: 15314	≥ 10 vs. 0	0.76 (0.68–0.86)		
Froehner 2014 ^[7]^	Germany	1993~2010	93.6	735	67.0	NR	>20 vs. < 10	0.63 (0.46–0.87)		
Simone 2013 ^[34]^	Italy	2002~2010	NR	933	66.3	≤ T2: 463 ≥ T3: 470	≥ 27 vs. < 27		0.73 (0.56–0.95)	0.70 (0.56–0.88)
Baumann 2013 ^[19]^	USA	1990~2008	44.1	442	65.8	≤ T2: 232 ≥ T3: 210	≥ 10 vs. < 10			0.37 (0.24–0.57)
Otto 2012 ^[30]^	Germany	1989~2008	42.0	2483	66.4	≤ T2: 1377≥ T3: 1107	>14 vs. ≤ 14		1.26 (0.96–1.67)	
Morgan 2012 ^[8]^	USA	1992~2006	NR	3170	75.0	≤ T2: 1158 ≥ T3: 2003	≥ 14 vs. 1-5	0.77 (0.66–0.90)	0.78 (0.65–0.93)	
Karadeniz 2011 ^[9]^	Turkey	1996~2009	20.0	74	61.7	≤ T2: 30 ≥ T3: 44	13-41 vs. 1-12	0.62 (0.46–0.84)	0.70 (0.49–1.00)	
Shirotake 2010 ^[26]^	Japan	1987~2008	64.0	169	68.0	≤ T2: 86 ≥ T3: 83	≥ 9 vs. < 9		0.29 (0.11–0.67)	
Hugen 2010 ^[20]^	USA	1996~2008	NR	260	66.9	≤ T2: 169 ≥ T3: 91	≥ 14 vs. < 14			0.78 (0.62–0.97)
Furukawa 2010 ^[27]^	Japan	1995~2003	NR	82	70.3	≤ T2: 19 ≥ T3: 63	≥ 10 vs. < 10		0.99 (0.56–1.75)	
Fang 2010 ^[21]^	USA	2000~2008	NR	349	66.0	≤ T2: 191 ≥ T3: 158	≥ 16 vs. 0-7	0.51 (0.30–0.85)		
Fairey 2009 ^[29]^	Canada	1994~2007	31.0	468	66.0	≤ T2: 230 ≥ T3: 238	≥ 11 vs. 0	0.74 (0.47–1.16)	0.94 (0.55–1.61)	
Kassouf 2008 ^[22]^	USA	1993~2003	24.0	248	NA	≤ T2: 57 ≥ T3: 191	>12 vs. ≤ 12		0.41 (0.29–0.58)	
Ide 2008 ^[28]^	Japan	1987~2003	42.0	146	67.0	≤ T2: 86 ≥ T3: 60	≥ 8 vs. < 8			0.19 (0.04–0.91)
Honma 2006 ^[10]^	Japan	1990~2002	35.0	146	65.0	≤ T2: 90 ≥ T3: 56	≥ 13 vs. < 13		0.11 (0.03–0.40)	
Lotan 2005 ^[23]^	USA	1984~2003	46.8	750	64.8	≤ T2: 441 ≥ T3: 309	>25 vs. < 13	0.66 (0.44–0.97)	0.44 (0.25–0.77)	0.39 (0.24–0.64)
Stein 2003 ^[24]^	USA	1971~1997	120.0	1054	66.0	NR	>15 vs. ≤ 15	0.55 (0.37–0.80)		0.73 (0.45–1.19)
Konety 2003 ^[25]^	USA	1988~1996	63.5	1923	64.3	≤ T2: 399 ≥ T3: 765	≥ 20 vs. 0		0.48 (0.30–0.76)	
Knap 2003 ^[35]^	Denmark	1992~1998	6.3	244	65.0	≤ T2: 205 ≥ T3: 39	≥ 12 vs. 1-3		0.80 (0.50–1.30)	

LND: Lymph node dissection; OS: Overall survival; CSS: Cancer-specific survival; RFS: Recurrence-free survival; NR: Not reported.

### Number of dissected nodes and OS of bladder cancer patients

**Figure 2:**
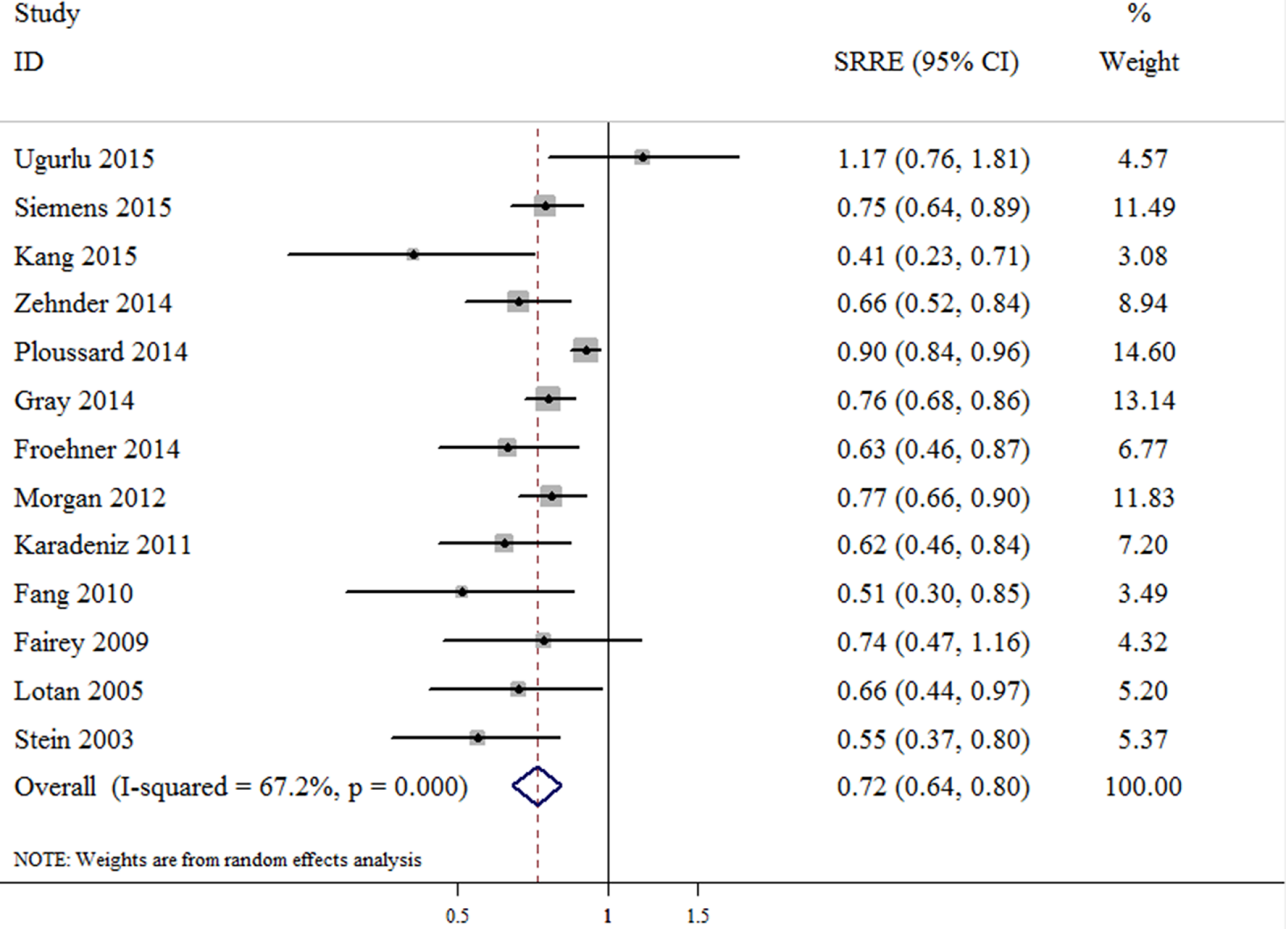
Meta-analysis of studies that examined the association between the number of dissected nodes and overall survival (OS) following radical cystectomy

Figure 2 shows the pooled results for the OS of bladder cancer patients using a random-effects model based on 13 studies, with a sample size of 34,128 individuals. An inverse association was identified between the highest vs. the lowest category of LND and the OS rates of bladder cancer patients treated with RC [summary relative risk estimate (SRRE) = 0.72; 95% CI, 0.64–0.80). Substantial heterogeneity was observed across studies (*p*-value for heterogeneity < 0.001; I^2^ = 67.2%; Figure 2). No statistical evidence of publication bias was indicated by the Begg's test (*p* = 0.161), but the Egger's test indicated the possible presence of publication bias (*p* = 0.003). Among the subgroup analyses, the majority of results were found to be consistent with the primary findings. However, no significant associations were identified in the models of OS stratified by mean age < 65 years (SRRE = 0.76; 95% CI, 0.53–1.11) or by studies conducted in Asia (SRRE = 0.68; 95% CI, 0.40–1.16) Table 2.

**Table 2:** Summary of meta-analysis results for the numbers of dissected lymph nodes and bladder cancer outcomes

Analysis specification	Highest category vs. lowest category			
	Studies	SRRE (95% CI)	p−het	I^2^
**Overall survival**				
All	13	0.72 (0.64−0.80)	0.000	67.2
Year of publication				
≥ 2011	9	0.75 (0.66−0.84)	0.000	71.7
< 2011	4	0.61 (0.49−0.76)	0.663	0.0
Sample size (cases)				
≥ 500	8	0.74 (0.67−0.83)	0.003	68.0
< 500	5	0.66 (0.48−0.91)	0.029	62.8
Mean age (years)				
≥ 65	10	0.72 (0.64−0.81)	0.000	69.8
< 65	3	0.76 (0.53−1.11)	0.052	66.2
Geographical region				
America	7	0.74 (0.69−0.80)	0.539	0.0
Europe	1	0.63 (0.46−0.87)	/	/
Asia	3	0.68 (0.40−1.16)	0.009	78.8
**Cancer-specific survival**				
All	14	0.66 (0.54−0.80)	0.000	73.3
Year of publication				
≥ 2011	6	0.79 (0.65−0.95)	0.011	66.6
< 2011	8	0.53 (0.37−0.75)	0.003	67.9
Sample size (cases)				
≥ 500	6	0.74 (0.59−0.93)	0.001	75.3
< 500	8	0.57 (0.41−0.80)	0.002	68.3
Mean age (years)				
≥ 65	10	0.75 (0.61−0.93)	0.001	69.1
< 65	3	0.56 (0.42−0.75)	0.271	23.5
Geographical region				
America	6	0.62 (0.49−0.79)	0.004	70.7
Europe	3	0.91 (0.62−1.34)	0.016	75.7
Asia	6	0.49 (0.29−0.84)	0.010	70.1
**Recurrence-free survival**				
All	9	0.64 (0.50−0.82)	0.000	72.5
Year of publication				
≥ 2011	5	0.67 (0.47−0.96)	0.000	80.1
< 2011	4	0.58 (0.37−0.89)	0.029	66.7
Sample size (cases)				
≥ 500	4	0.65 (0.53−0.80)	0.158	42.3
< 500	5	0.59 (0.34−1.03)	0.000	82.9
Mean age (years)				
≥ 65	7	0.63 (0.51−0.78)	0.038	55.0
< 65	2	0.74 (0.21−2.55)	0.000	93.2
Geographical region				
America	5	0.53 (0.36−0.77)	0.006	72.3
Europe	1	0.70 (0.56−0.88)	/	/
Asia	2	0.59 (0.09−4.06)	0.016	82.7

SRRE: summary relative risk estimate.

### Number of dissected nodes and CSS of bladder cancer patients

**Figure 3:**
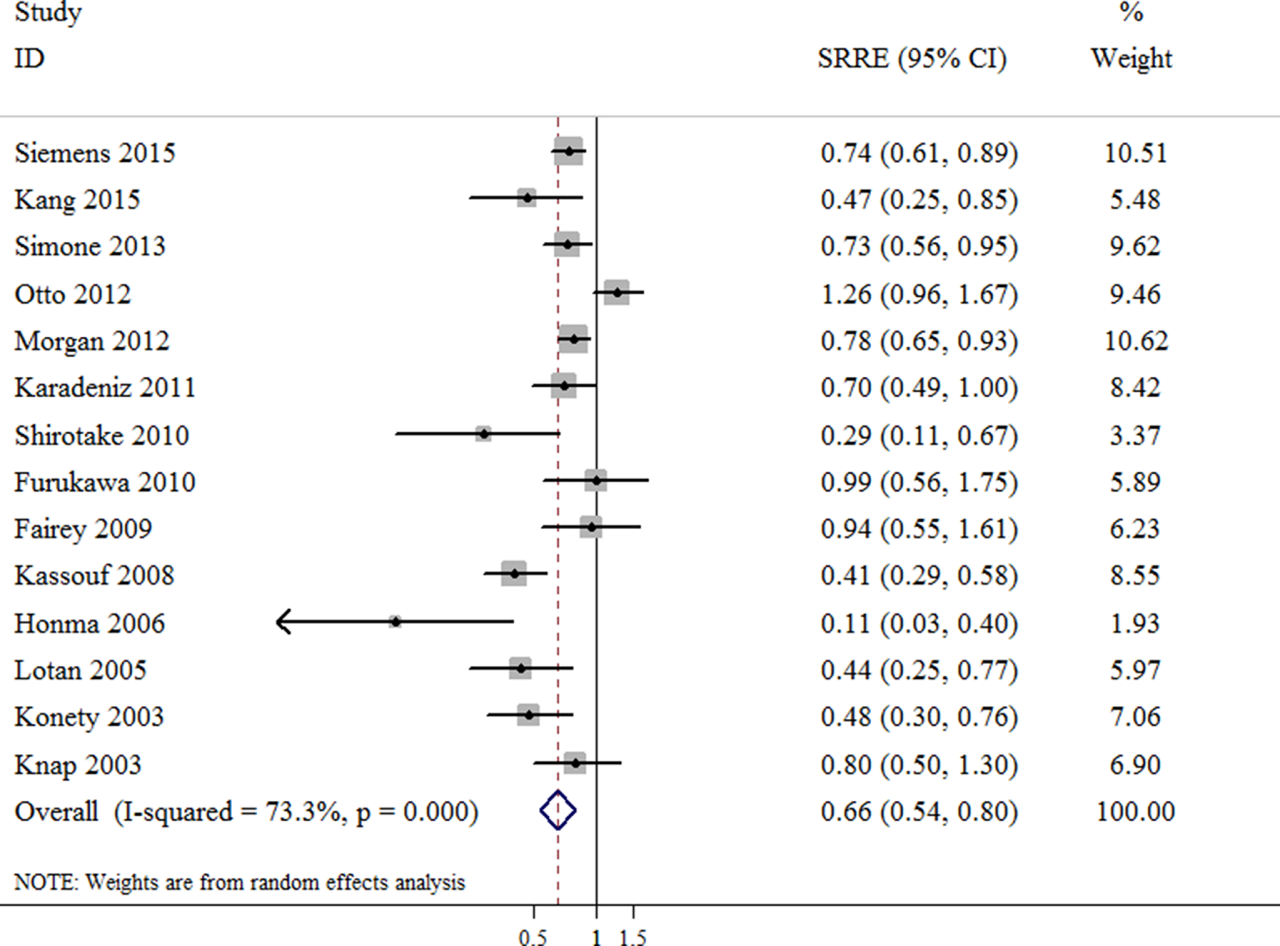
Meta-analysis of studies that examined the association between the number of dissected nodes and cancer-specific survival (CSS) following radical cystectomy

The outcomes of bladder cancer were presented as CSS in 14 studies with a total of 12,518 bladder cancer patients. A decreased risk, corresponding to a higher CSS rate of bladder cancer patients was found in the patients in whom a greater number of nodes were removed during RC (SRRE = 0.66; 95% CI, 0.54–0.80; Figure 3), with evidence of heterogeneity observed (*p*-value for heterogeneity < 0.001; I^2^ = 73.3%). There was no statistical evidence of publication bias among the studies indicated by the Begg's or Egger's tests (Begg, *p* = 0.189; Egger, *p* = 0.072). Furthermore, inconsistent pooled results were found in the meta-analysis of CSS stratified by studies conducted in Europe (SRRE = 0.91; 95% CI, 0.62–1.34) Table 2.

### Number of dissected nodes and RFS of bladder cancer patients

**Figure 4:**
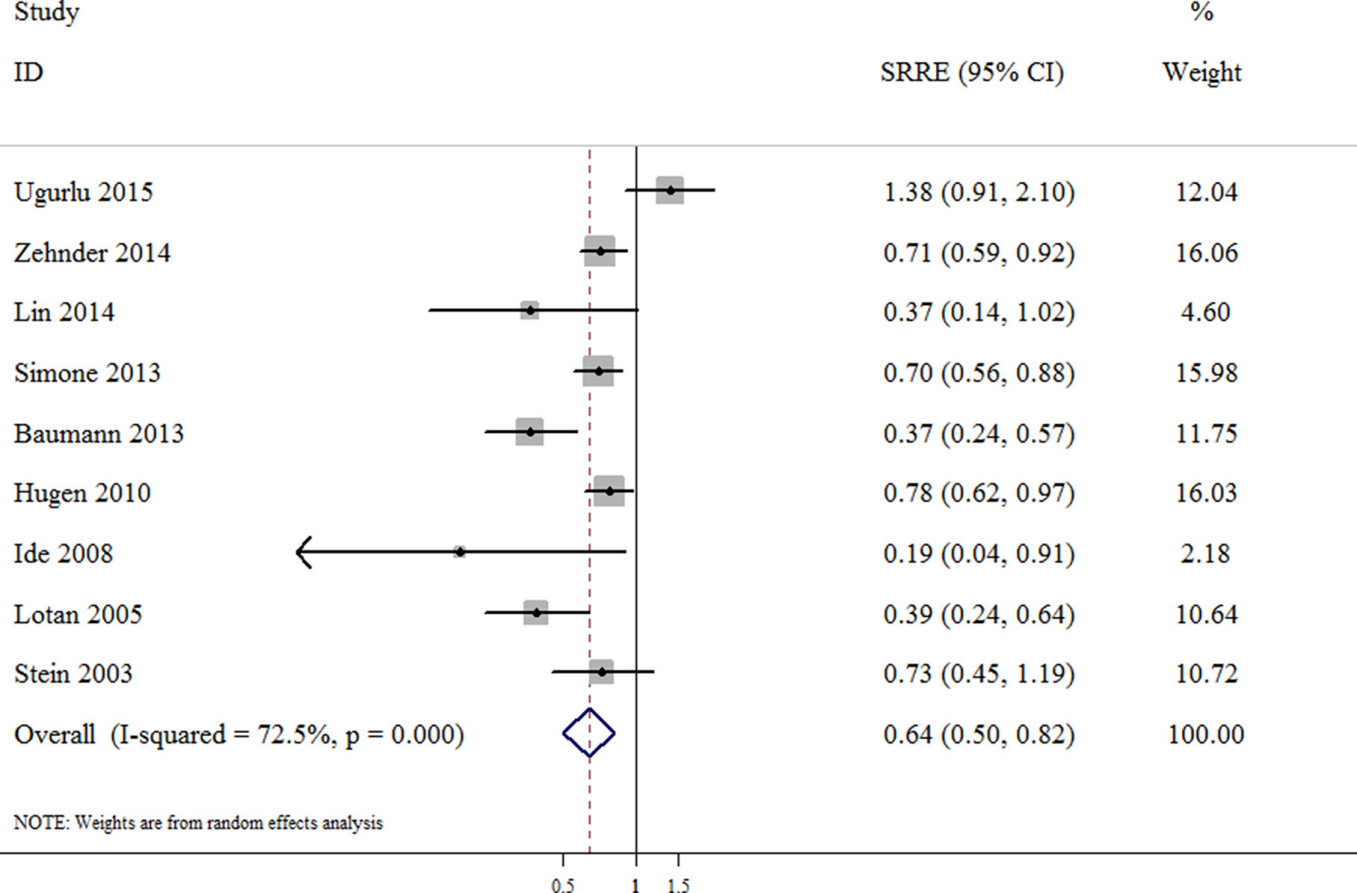
Meta-analysis of studies that examined the association between the number of dissected nodes and recurrence-free survival (RFS) following radical cystectomy

The association between the number of dissected nodes and the RFS of bladder cancer patients following RC was reported in 9 studies, including 4,387 bladder cancer patients. The summarized result of these studies indicated that a significant inverse association was observed between the highest vs. the lowest category of LND and the RFS of bladder cancer following RC, with an SRRE of 0.64 (95% CI, 0.50–0.82; Figure 4), and evidence of heterogeneity being found among the studies (*p*-value for heterogeneity < 0.001; I^2^ = 72.5%). The Begg's (*p* = 0.175) and Egger's tests (*p* = 0.224) provided no evidence of a significant publication bias among the studies on RFS. In addition, no significant association was also observed among studies of RFS when restricted to studies with a sample size of < 500 patients (SRRE = 0.59; 95% CI, 0.34–1.03), a mean age of < 65 years (SRRE = 0.74; 95% CI, 0.21–2.55), or studies conducted in Asia (SRRE = 0.59; 95% CI, 0.09–4.06) Table 2.

### Sensitivity analyses

Sensitivity analyses were performed by sequentially excluding each study in turn to examine the influence of individual studies on the overall or stratified estimates. None of the studies considerably affected the pooled effects observed in the meta-analysis (data not shown).

## DISCUSSION

Approximately 35% of patients succumb to disease following RC. The aim of RC is the complete eradication of local and regional disease, which is achieved by excision of the bladder, perivesical soft tissues, adjacent organs and regional lymph nodes [34]. Therefore, a number of different variables may affect survival outcomes following surgical intervention; these variables include lymph node status, surgical margin status, pathological stage and the number of lymph nodes dissected [35]. Although many studies have demonstrated that statistically significant survival advantages may be observed among groups of patients with a greater number of dissected nodes during RC [6, 7, 9, 10], other studies have indicated that there is no significant association between the two [4, 14, 24]. Thus, reports in the literature regarding the correlation between the number of dissected nodes and the prognosis following RC are conflicting.

A substantial proportion of bladder cancer patients will have microscopic systemic spread at the time of surgery [35]. Of these patients, removing additional lymph nodes outside of the standard area will cure only a minority. Therefore, the therapeutic effects attained by extended LND are likely to be relatively small (if they exist), and studies with extremely large sample sizes are required to verify this. Certain biases (for instance, removing more lymph nodes in younger, healthier patients with early-stage disease) may result in great variability in outcome [10]. Remarkably, the survival outcomes reported in one study comparing extended with super-extended dissection were not better than in comparable series using standard dissection [36].

To the best of our knowledge, the present meta-analysis was the first to explore the potential relationship between the number of nodes removed at LND and the outcomes of bladder cancer patients treated with RC. We pooled 25 cohort studies, with a large sample size of ~41,400 bladder cancer patients who underwent RC, in order to enhance the statistical power available to estimate the overall associations, and obtain a stable and credible result. Quantitative analysis of the published epidemiological studies indicated that the bladder cancer patients with a greater extent of LND during RC had statistically significant advantages in terms of OS, CSS and RFS, corresponding to reduced risks of 28%, 34% and 36%, respectively, compared with patients with a lesser extent of LND.

A large degree of heterogeneity was observed across studies included in this analysis. However, an absence of publication bias in these meta-analyses was shown using the Egger's and Begg's tests; one exception was the result of the Egger's test in the model of OS, which suggested possible publication bias. In addition, sensitivity analyses yielded similar and robust results, indicating that no study considerably affected the summary associations between the extent of LND and the outcomes of bladder cancer patients who underwent RC.

Heterogeneity is a significant concern in meta-analyses. Substantial heterogeneity was detected across the included studies, which may reflect differences in the study populations, analytical methodology and adjustment for confounding factors. Although subgroup analyses were performed to examine potential sources of heterogeneity, stratifying studies by mean age, sample size, study design, year of publication and geographical region, the possible source of heterogeneity was not identified. Differences in LND template, tissue submission technique, pathological evaluation and lymph node identification may lead to heterogeneity between the individual studies. The subgroup analyses based on the above four issues were not conducted due to limited data. In addition, we could not exclude the possibility of various other unknown factors contributing to the variability.

Concerning the significant inverse associations between the number of lymph nodes dissected and the outcomes of bladder cancer following RC, one plausible explanation is that removal of more lymph nodes improves the thoroughness of the pathology evaluation, which may be beneficial in guiding more accurate disease staging and subsequent therapy [9, 15]. The findings underline the necessity for guidelines regarding surgical lymphadenectomy and the pathological assessment of lymph nodes in bladder cancer. Among the subgroup analyses, we identified inconsistent findings in certain subgroups, such as studies with a sample size of < 500, studies reporting a mean age of < 65 years, or those conducted in Asia or Europe. The reason for these inconsistent findings is uncertain. Possible explanations may be the limitations of the studies, different techniques used by surgeons or pathologists, different tumor stages, differences in subsequent therapies, adjusted factors, dietary factors, or other unmeasured or unknown factors [8, 37].

The present study has several important limitations that must be taken into account when considering its contribution to the field. First, and most significantly, are the limitations inherent to retrospective analyses, which accounted for the majority of included studies; studies with this design are susceptible to bias and may thus produce results that are difficult to interpret. Although there were some prospective cohort studies that met our inclusion criteria, they comprised a relatively small number of patients. Secondly, substantial heterogeneity was observed among the studies, although the possible sources of heterogeneity were not identified despite the conduction of several subgroup analyses. The pooled results in the majority of subgroup analyses were consistent the overall findings. Furthermore, our sensitivity analyses indicated the robustness of the current findings. Thirdly, the bladder cancer patients in the included studies underwent RC by multiple different surgeons, and their specimens were evaluated by different pathologists; variation in such practices could affect accurate clinical staging. Stage discrepancy remains a significant problem across all analyzed treatment facilities and affects the summary associations [38]. Furthermore, this variability may have contributed to the observed heterogeneity in this meta-analysis. However, this may also be regarded as a strength as it represents real-world practice.

In summary, the present analysis summarizes the available evidence that statistically significant advantages in the OS, CSS and RFS of bladder cancer may be achieved by the dissection of a greater number of lymph nodes during RC. The extent of LND may be an independent prognostic factor for bladder cancer. These findings must be validated in prospective and larger epidemiological studies with longer follow-up periods.

## MATERIALS AND METHODS

### Search strategy

The present meta-analysis was performed in accordance with the Preferred Reporting Items for Systematic Reviews and Meta-analyses (PRISMA) guidelines [39]. A systematic literature search was performed in PubMed, Embase and the Cochrane Library to identify the eligible studies published from the inception of the databases to April 2016. The primary search string included the following items: ‘radical cystectomy’, or ‘bladder cancer’, or ‘transitional cell carcinoma’, or ‘urinary bladder neoplasms’; ‘lymphadenectomy’, or ‘lymph node dissection’, or ‘lymph node excision’, or ‘lymph node removed’; ‘outcome’, or ‘survival’, or ‘mortality’, or ‘recurrence’. Our search focused on human studies, without a restriction on language. In addition, the reference lists of all included articles we reviewed to identify additional available studies.

### Inclusion and excluded criteria

The eligibility of each study was assessed by the population, intervention, comparator, outcome and study design (PICOS) approach [39]. Studies were included in the meta-analysis if they met the following criteria: the bladder cancer patient was treated with RC (P); the LND was performed during RC (I); the numbers of lymph nodes removed were evaluated (C); risk estimates [hazard ratios, risk ratios, odd ratios] with corresponding 95% CIs were reported, or sufficient data were provided to estimate these (O); the study design was a prospective or retrospective cohort study (S).

In addition to these criteria, the survival outcomes of bladder cancer were defined as ‘overall survival (OS)’, ‘cancer-specific survival (CSS)’, and ‘recurrence-free survival (RFS)’, and these were synthesized respectively in the meta-analysis. The exclusion criteria were as follows: i) duplicates; ii) no usable data reported; iii) case-reports, reviews, expert opinions or meeting abstracts, cross-sectional, case-control and ecological analyses. In cases of more than one publication using the same or an overlapping cohort, only the most recent and informative one was included.

### Data extraction

Two of the authors independently extracted the information from the selected studies using a standardized data collection form. Discrepancies that arose were resolved by repeating the review of the studies and the discussion. The following information was extracted: first author, year of publication, study design, study location, study period, duration of follow-up, sample size, mean age, gender, pathological stages, positive lymph node rate, the number of dissected nodes, and risk estimates of OS, CSS or RFS based on the highest vs. the lowest categories of the extent of LND. If a study reported multiple data sets, the results from the main multivariable model that included the most adjusted confounders were used.

### Statistical analysis

The SRRE for the highest vs. the lowest category of LND was used. Most of the included studies used Cox proportional hazard ratio models to analyze the number of dissected nodes with regard to the survival outcomes of bladder cancer. From these studies, we used the reported hazard ratios and 95% CIs, or the reciprocally converted values for calculation. For studies that used the Kaplan-Meier method and log-rank test to estimate survival, the risk estimates and 95% CIs were calculated according to method described by Parmar *et al.* [40] and Altman *et al.* [41].

Fixed- and random-effects methods were both used in order to estimate the association between outcomes of bladder cancer treated with RC and the highest category of number of lymph nodes removed vs. the lowest category. Statistical heterogeneity among the studies was assessed using the Q statistic (with *P* < 0.10 used as the threshold for significance). The I^2^ statistic was also calculated in order to quantitatively assess the inconsistency across studies, with values of 75, 50 and 25% defined as representing high, medium and low degrees of heterogeneity, respectively. In addition, forest plots were constructed to evaluate the associations between the extent of LND and the various survival outcomes of bladder cancer treated with RC.

Subgroup analyses were performed to examine potential sources of heterogeneity according to the year of publication (≥ 2011 vs. < 2011), sample size (≥ 500 vs. < 500 patients), mean age (≥ 65 vs. < 65 years) and geographical region (America, Europe and Asia). Sensitivity analyses were also conducted to assess the robustness of the results, by repeating the meta-analysis after omitting one study at a time. Furthermore, Egger's test and Begg's method were applied to evaluate the possible bias, combined with a visual inspection of the funnel plot. All statistical analyses were conducted using STATA 12.0 (StataCorp LP, College Station, TX, USA). A two-tailed *P* value of < 0.05 was considered to indicate statistical significance, except where specifically noted.
